# From laurels to parasites: the origin, evolution, systematics, and applications of *Cassytha* (Lauraceae)

**DOI:** 10.3389/fpls.2026.1790129

**Published:** 2026-03-19

**Authors:** Zhi-Fang Liu, Jie Li, John G. Conran

**Affiliations:** 1 Institute of Leisure Agriculture, Shandong Academy of Agricultural Sciences, Jinan, China; 2 Shandong Engineering Research Center of Ecological Horticultural Plant Breeding, Jinan, China; 3 Plant Phylogenetics and Conservation Group, Center for Integrative Conservation & Yunnan Key Laboratory for Conservation of Tropical Rainforests and Asian Elephants, Xishuangbanna Tropical Botanical Garden, Chinese Academy of Sciences, Mengla, Yunnan, China; 4 Environment Institute, School of Biological Sciences, Adelaide University, North Terrace, Adelaide, SA, Australia

**Keywords:** biological control, *Cassytha*, evolution of parasitism, lauraceae, medicinal plants, parasitic plants

## Abstract

*Cassytha*, an obligate stem-parasitic genus within the laurel family (Lauraceae), represents a remarkable evolutionary transition. It retains partial photosynthetic capacity and key morphological traits of its woody relatives, positioning it as a key model for understanding the early evolutionary stages of parasitism. This review synthesizes current knowledge on its origin, parasitic adaptations, and phylogenetic placement. *Cassytha* apparently evolved from free-living woody ancestors within Lauraceae and retains some of its photosynthetic ability, serving as a key model for studying the basal lineage of parasitic evolution. However, its taxonomy remains problematic due to morphological reduction and high variability. Biogeographic evidence suggests a Southern Hemisphere origin, with buoyant fruits facilitating its dispersal. Beyond its scientific interest, *Cassytha* holds applied value as a source of pharmacologically active alkaloids and as a potential biocontrol agent against invasive plants. Future research should clarify its species boundaries, elucidate the genetic basis of parasitism, and assess the ecological risks of its practical use.

## 1 Introduction

The laurel family (Lauraceae) comprises approximately 50 genera and about 3,000 species of mostly woody, aromatic trees and shrubs distributed worldwide, particularly in tropical and subtropical regions (Rohwer, 1993; van der Werff and Richter, 1996; Chanderbali et al., 2001; Li et al., 2025). Members of the family are ecologically dominant in many forest ecosystems and economically important as sources of timber, spices, essential oils, and medicinal compounds. Against this backdrop of morphological and ecological conservatism, *Cassytha* Osbeck represents a striking evolutionary anomaly. Unlike its free-living relatives, *Cassytha* consists of obligate stem parasites characterized by filamentous, twining stems, highly reduced leaves, and specialized haustoria that penetrate host tissues. Often referred to as “dodder-laurels” because of their superficial resemblance to *Cuscuta* L. (Convolvulaceae), *Cassytha* species are phylogenetically unrelated to true dodders.

The evolution of parasitic plants involved multiple independent transitions from autotrophy to parasitism, occurring ca. 12–13 times from autotrophic plants (Westwood et al., 2010; Nickrent, 2020). *Cassytha* represents one of the few independent origins of stem parasitism outside the well-studied root parasitic groups (e.g., Orobanchaceae) and the independently evolved stem parasite *Cuscuta* (Convolvulaceae). Despite its evolutionary significance, *Cassytha* has received disproportionately limited research attention: a Web of Science search (February 2026) yields approximately 136 publications focusing on *Cassytha*, compared with over 1,200 for *Cuscuta* and more than 1,000 for Orobanchaceae. This disparity highlights the need for synthesis and renewed investigation into this understudied parasitic lineage.

Studying stem parasitism, as exemplified by *Cassytha*, offers distinct insights compared with root parasitism. Stem parasites interface directly with host above-ground tissues, facing different physiological challenges and ecological interactions than root parasites, which access hosts below-ground. Unlike root parasites that typically connect to host xylem, stem parasites like *Cassytha* must contend with host defense responses in photosynthetic tissues and compete directly for light and space. Furthermore, the phylogeny relationships of *Cassytha* within a predominantly non-parasitic family (Lauraceae) provide a unique opportunity to investigate the early evolutionary steps toward parasitism, including the retention of photosynthetic capacity and the gradual acquisition of haustoria function, features that are largely lost in more derived parasites.

It is worth noting that while a previous review (Zhang H. et al., 2022) comprehensively examined the parasitic habits, host range, and ecological interactions of *Cassytha*, the present review adopts a distinct and complementary focus. Specifically, we emphasize the evolutionary origin, phylogenetic placement, and morphological adaptations of *Cassytha*, along with its dual applied potential as a source of bioactive alkaloids and as a biocontrol agent against invasive plants.

Despite recent advances in angiosperm phylogenetics and parasitic plant biology, fundamental questions regarding *Cassytha* remain unresolved. Its species boundaries are poorly defined due to morphological reduction and cryptic diversity, and the genetic mechanisms underlying its transition from woody ancestors to hemiparasitism have yet to be elucidated. Furthermore, while *Cassytha* shows promise as a source of bioactive compounds and as a biocontrol agent, these applied potentials have not been critically evaluated in light of recent findings. A comprehensive synthesis is particularly timely now because recent phylogenomic studies have clarified the position of *Cassytha* within Lauraceae and revealed unexpected cryptic diversity (Liu et al., 2024), while growing interest in parasitic plants as evolutionary models and their emerging applications in ecological management and drug discovery create a need to integrate disparate lines of evidence. This review, therefore, synthesizes current knowledge across evolutionary biology, systematics, biogeography, and applied research to provide a foundation for future investigations into this unique parasitic lineage.

The literature search strategy for this review is provided in the **Supplementary Material**.

## 2 Parasitic evolution and systematics of *Cassytha*

### 2.1 Ancestral state and evolutionary origins

**Figure 1:**
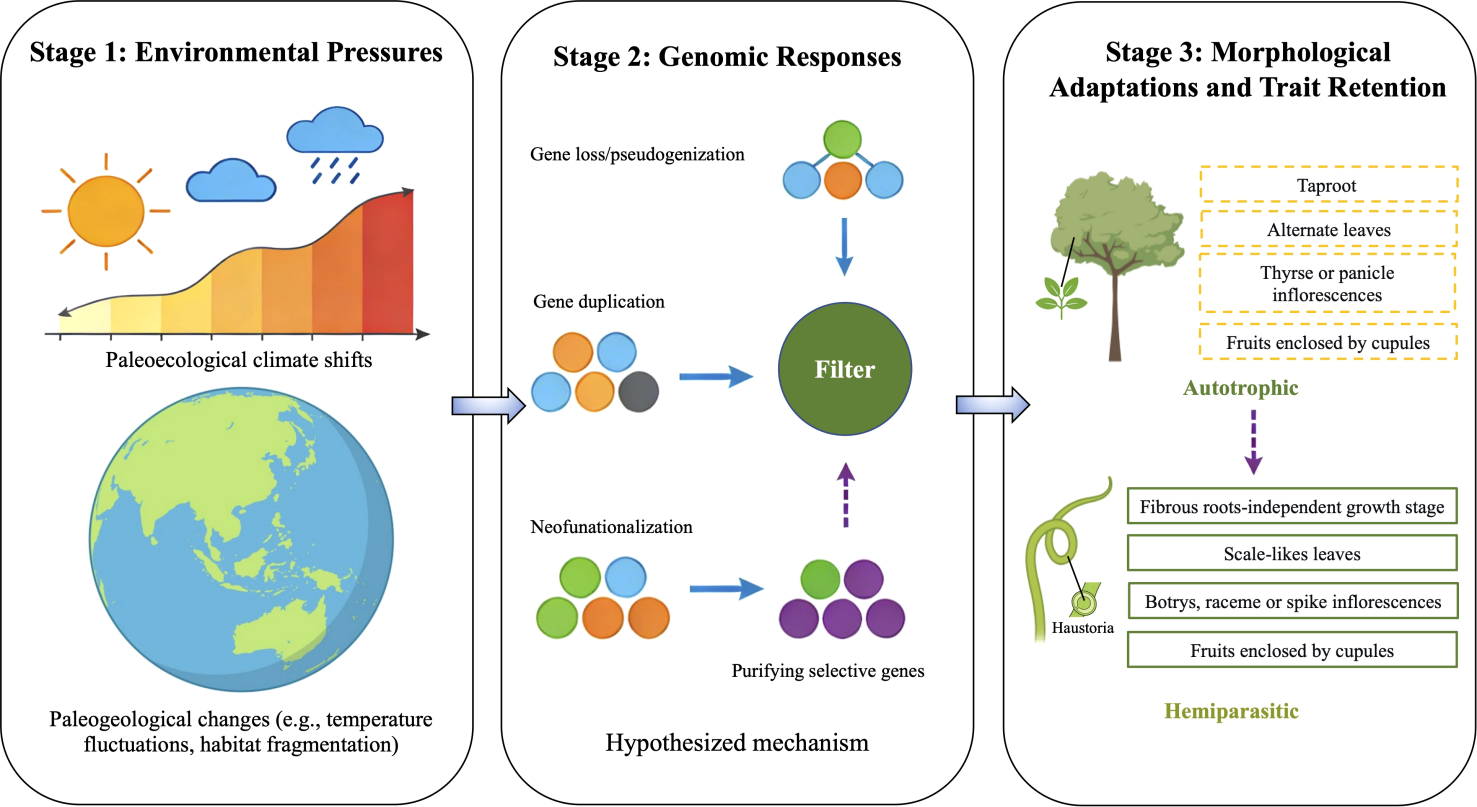
A schematic diagram illustrating the hypothesized evolutionary pathway of *Cassytha*. The proposed model integrates phylogenetic evidence with inferred morphological and genomic changes tree sequential stages: (1) Environmental pressures (paleoecological climate shifts and paleogeological changes) act as extremal drivers; (2) Genomic responses include gene losses/pseudogenization, hyper-purifying selection that maintains essential and reproductive genes; (3) Morphological adaptations (twining stems, haustoria development) occur while ancestral Lauraceae floral and fruit trats are retained. Solid arrows indicate empirically supported relationships; dashed arrows represent hypothesized connection hypotheses, proposed here for the first time, integrating phylogenetic evidence with inferred morphological and genomic changes. Graphic elements were created using Doubao (https://www.doubao.com) and BioRender (https://www.biorender.com/).

The evolutionary pathway leading to the twining hemiparasitic habit in *Cassytha* remains incompletely understood (Westwood et al., 2010; Nickrent, 2020; Zhang H. et al., 2022). Unlike many advanced parasitic plants that have lost photosynthetic capacity entirely (Westwood et al., 2010), *Cassytha* retains functional chloroplasts and can perform photosynthesis, albeit at reduced levels. This suggests that *Cassytha* represents an intermediate evolutionary stage between autotrophy and full parasitism: it is an obligate parasite (requiring a host to complete its life cycle) but retains hemiparasitic capacity (functional photosynthesis), unlike holoparasitic plants that have completely lost photosynthetic ability (Westwood et al., 2010). This dual dependence on both host-derived resources and self-produced photosynthesis positions *Cassytha* as a key model for understanding the early stages of parasitic plant evolution. Analyses of plastid and nuclear DNA sequences consistently recover *Cassytha* as a monophyletic group nested among the otherwise woody Lauraceae, sister to certain woody genera such as *Neocinnamomum* H. Liu and *Caryodaphnopsis* Airy Shaw (Chanderbali et al., 2001; Rohwer and Rudolph, 2005; Li et al., 2016; Liu et al., 2021). Here we hypothesize that *Cassytha* is derived from free-living, woody ancestors within Lauraceae (**Figure 1**). This hypothesis is inferred from its phylogenetic relationships and the morphological traits of its closest extant relatives within Lauraceae, such as *Neocinnamomum* and *Caryodaphnopsis*. Driven by paleoecological and paleogeological changes, the ancestral woody lineage underwent genomic alterations associated with the shift to parasitism. Plastid genome comparisons have revealed gene loss and pseudogenization in *Cassytha filiformis* L., with *dN/dS* analyses indicating that retained genes remain under strong purifying selection (Wu et al., 2017; Lin et al., 2022). We hypothesize that gene duplication and neofunctionalization, mechanisms known in other parasitic plants (Yang et al., 2015; Xu et al., 2022), may also have contributed to parasitic adaptations in *Cassytha*, although this remains to be confirmed. Morphological changes accompanying parasitism include reduction of leaves to scales, development of twining stems, and formation of haustoria. Meanwhile, purifying selection-maintained genes essential for reproductive structures (e.g., flowers and fruits), enabling *Cassytha* to retain key lauraceous traits and its consistent phylogenetic placement within the family (**Figure 1**).

There is currently no fossil record of *Cassytha*, so its origin time can only be inferred based on the fossil records of other Lauraceae. Previous studies suggest that the stem age of *Cassytha* dates to ca. 88 Ma (Li et al., 2016; Liu et al., 2024). The timescale for the divergence between *Cassytha* and *Neocinnamomum* was estimated to range from 92.6–62.6 Ma (median 74 Ma), but the adjusted time is 46 Ma (http://www.timetree.org) (Kumar et al., 2017). The discrepancies in the estimated divergence times for *Cassytha* could be attributed to several factors. Different studies often employ varying methodological approaches and calibration points, such as distinct fossil selections or molecular clock models. Furthermore, integrative databases like TimeTree periodically refine estimates by incorporating broader datasets and more complex statistical models (Kumar et al., 2017). Differences in genetic markers or taxonomic sampling completeness between earlier and subsequent studies may also lead to revised estimates (Massoni et al., 2015; Li et al., 2019; Ramírez-Barahona et al., 2020; Zhang H. et al., 2022). Such divergence in dates reflects the iterative nature of scientific inference, where estimates are continually updated with new evidence and analytical improvements. Liu et al. (2024) estimated the crown age of the genus to be ca. 37 Ma, but that study only included three *Cassytha* species, and their estimates, based on complete chloroplast genomes, are inconsistent with those derived from nrDNA data. Unlike more highly derived parasites, *Cassytha* retains photosynthetic ability and a relatively complete genome, making it an ideal system for studying the initial genetic and developmental changes associated with the evolution of parasitism.

### 2.2 Morphological, chemical, and systematic evidence for lauraceous affinity

The vegetative structure of *Cassytha* is highly reduced, reflecting its parasitic habit. Leaves are scale-like, while stems are slender, filiform, and twining, enabling the plant to climb and attach to hosts (Weber, 1981; 2007). Haustoria develop at points of contact with host stems, penetrating the vascular tissue to establish a physiological connection. The haustoria of *Cassytha* primarily connect to the host xylem to extract water and minerals, but lack direct phloem connections, which may explain the retention of partial photosynthetic capacity. This xylem-feeding habit represents an intermediate stage in the evolution of obligate parasitism, as hemiparasites that access only host xylem are considered evolutionarily transitional between fully autotrophic plants and holoparasites that also tap host phloem (Westwood et al., 2010; Nickrent, 2020).

Despite its parasitic habit, *Cassytha* exhibits a suite of diagnostic lauraceous characters in floral and fruit morphology, as well as in stem and leaf anatomy (Rohwer, 1993). These include small, actinomorphic flowers with six tepals arranged in two whorls of three, nine fertile stamens, typically in three whorls, paired glands on the third stamen whorl, valvate anther dehiscence, a single superior ovary with one ovule, and lauraceous ovule development (Weber, 1981, 2007; Liu et al., 2024). Besides, *Cassytha* shares several anatomical features with other Lauraceae, including the presence of oil cells and mucilage canals, which are considered synapomorphic for the family (Rohwer, 1993).

Chemotaxonomic analyses reveal that *Cassytha* produces characteristic Lauraceae secondary metabolites, including benzylisoquinoline alkaloids, lignans, and essential oils (Gottlieb, 1972). These chemical markers provide independent support for the placement of the genus within the family.

The independent origin of parasitism in *Cassytha* demonstrates that major ecological transitions can occur within morphologically conservative families. Its evolutionary trajectory highlights the role of genomic plasticity and modularity in enabling radical ecological shifts without complete loss of ancestral traits. The retention of ancestral lauraceous characters alongside derived parasitic adaptations makes *Cassytha* a valuable model for understanding the interplay between phylogenetic constraint and ecological innovation. Research on haustorial development, host recognition, and nutrient transfer in *Cassytha* can provide insights into the mechanisms of plant, plant interactions and parasitic adaptation.

## 3 Taxonomic challenges

The taxonomy within *Cassytha* is complicated and contentious due to the scarcity of reliable taxonomic characters. The taxonomy of *Cassytha* remains problematic due to a lack of reliable diagnostic characters and limited revisionary work. The genus comprises approximately 20 species (Weber, 1981, 2007), but species boundaries are poorly resolved, largely owing to morphological reduction and the absence of modern taxonomic synthesis.

Recent molecular studies have begun to clarify the extent of this uncertainty. Liu et al. (2024) analyzed sequences from over 100 individuals and found that while most species formed well-supported clades, some relationships stayed unresolved. More strikingly, dense sampling of widespread *C. filiformis* revealed two deeply divergent lineages with distinct traits, indicating hidden cryptic diversity (Liu et al., 2024). These findings suggest that current classifications substantially underestimate species-level diversity and that morphological convergence has masked evolutionary lineages.

Nomenclatural issues further complicate taxonomy. Several species were described solely from herbarium specimens, often lacking critical reproductive structures, and type materials are incomplete for some names (Weber, 1981). Geographic variation in traditionally used traits, such as pubescence, adds further uncertainty, as both glabrous and hairy forms occur within the same species across their ranges (Weber, 1981, 2007; Liu et al., 2021).

Taxonomic ambiguity has significant implications beyond systematics. In ecological research, accurate species identification is critical for interpreting host range, distribution, and community interactions. For applied fields such as pharmacology and biocontrol, cryptic lineages may differ in alkaloid profiles or host specificity, affecting both efficacy and safety. The cryptic diversity uncovered in *C. filiformis* (Liu et al., 2024) underscores the need for lineage-specific assessments in future studies. Integrative approaches combining morphology, genomics, ecology, and chemistry are urgently needed to revise the genus and support future research.

## 4 Biogeography and ecological adaptation

### 4.1 Distribution patterns

*Cassytha* exhibits centers of diversity in Australia (ca. 20 spp.), a few areas in Africa, Southeast Asia, and the Pacific islands, based on the information records in GBIF (https://www.gbif.org/). With precipitation, temperature, and soil as the main limiting factors, the MaxEnt model demonstrated high accuracy in predicting the potential distribution areas of *Cassytha* (Zhang, 2024). Biogeographic reconstructions suggest a Southern Hemisphere origin, possibly associated with Gondwanan vicariance, followed by later dispersal and regional diversification. Several *Cassytha* species are restricted in range and may be threatened by habitat loss and climate change. Understanding their taxonomy, ecology, and genetic diversity is essential for conservation planning.

### 4.2 Dispersal mechanisms

The buoyant fruits of *Cassytha*, particularly *C. filiformis*, facilitate long-distance oceanic dispersal, as they can float for months, helping to explain its widespread pantropical coastal distribution (Muir, 1933). *Cassytha* has refractory (hard-coated) seeds and is found predominantly in coastal regions, a distribution pattern consistent with water dispersal (Mahadevan and Jayasuriya, 2013). Birds and humans also contribute to local and regional dispersal due to their fleshy fruit (Westoby et al., 1996), and the wide range of host plants further enhances its colonizing success (Zhang H. et al., 2022). These traits not only explain its wide distribution but also contribute to its capacity to interact strongly with native and invasive plant communities.

## 5 Application of *Cassytha*

### 5.1 Medicinal uses and phytochemical potential

*Cassytha* holds a significant place in various traditional medicine systems globally, including Ayurvedic, Chinese, and African practices, where they have been employed to treat a wide spectrum of ailments (Adamu et al., 2017; Zhang H. et al., 2022). These applications historically encompass the treatment of conditions such as cancer, sleeping sickness, malaria, urinary disorders, nephritis, headaches, hepatitis, piles, and sinusitis (Adamu et al., 2017; Zhang Q. et al., 2022; Tchamba et al., 2024). The empirical basis for these traditional uses is increasingly supported by modern scientific investigation, which has identified antipyretic and analgesic properties in *Cassytha* (Rajamanickam et al., 2022). Furthermore, bioactive compounds, particularly aporphine alkaloids, have been isolated and linked to specific pharmacological activities, including antitrypanosomal, cytotoxic, hypoglycemic, and antiplatelet effects (Sagar et al., 2025). Moreover, extending beyond these traditionally targeted conditions, specific alkaloids are under investigation for their neuroprotective potential (Cava et al., 1968; Rajamanickam et al., 2022). Their potent inhibitory action on glycogen synthase kinase 3 (GSK-3) suggests promising avenues for future research into managing neurodegenerative diseases like Alzheimer’s (Khabiya et al., 2024). In addition to alkaloids, *Cassytha* species produce essential oils, similar to other Lauraceae, with potential pharmacological applications (Brophy et al., 2009). Flavonoids have also been identified in *Cassytha*, contributing to its antioxidant and anti-inflammatory properties (Yokota, 2008). Furthermore, some aporphine alkaloids from *Cassytha* exhibit antitrypanosomal activity and can interact with DNA as intercalating agents (Hoet et al., 2004). *Cassytha* represents a valuable reservoir of bioactive compounds, providing a compelling scientific rationale for its extensive use across multiple traditional medicine systems and underscoring its promise for future drug discovery.

Caution is warranted in medicinal use, as *C. filiformis* may absorb toxic alkaloids, such as gelsemine, from its host plants like *Gelsemium* Juss. species, leading to potential human toxicity (Cheung et al., 2018). Therefore, the host plant source should be considered when harvesting *Cassytha* for medicinal purposes.

### 5.2 *Cassytha* in ecological management and biological control

Recent research highlights the significant potential of *Cassytha* species in ecological management and biological control. Studies in southern Australia have demonstrated that the native *C. pubescens* R.Br. can effectively suppress alien invasive leguminous shrubs such as *Ulex europaeus* L. and *Cytisus scoparius* (L.) Link (Facelli et al., 2020; Cirocco et al., 2021), supporting its role as a promising biocontrol agent in the region. Furthermore, the congeneric *C. filiformis* has shown notable parasitic efficacy against another invasive alien species in China, *Mikania micrantha* Kunth (Asteraceae), a fast-growing vine responsible for widespread ecological and economic damage (Liu et al., 2023). These findings collectively underscore the capacity of *Cassytha* to function as a natural regulator of invasive plant populations. *Cassytha* species can significantly reduce host growth, photosynthetic capacity, and reproductive output. While this parasitism can be detrimental in agricultural or forestry contexts, it may be advantageous in natural and semi-natural ecosystems where dominant or invasive species require control. Compared with chemical control, the use of a native or naturalized parasitic plant offers a more sustainable and ecologically integrated approach.

Despite its promise, the application of *Cassytha* in biological control requires careful risk assessment. Its broad host range raises concerns about non-target effects, particularly on native or economically valuable plants. Future research should therefore focus on host specificity, population genetics, and controlled field trials to evaluate both efficacy and safety.

## 6 Conclusion and future perspectives

*Cassytha* is a rare stem parasite that retains photosynthesis, providing a model for early parasitism evolution. Taxonomic uncertainty, including cryptic diversity, complicates its conservation and application. Pharmacological studies reveal bioactive alkaloids with therapeutic potential, while biocontrol trials show efficacy against invasives, yet risks from toxin accumulation and non-target effects persist. Future integrated research should resolve species boundaries, elucidate parasitic mechanisms, and validate applications through *in vivo* and field studies.
