# Supplementary material for: From laurels to parasites: the origin, evolution, systematics, and applications of Cassytha (Lauraceae)
Source: Front Plant Sci. 2026 Mar 19;17:1790129. doi: 10.3389/fpls.2026.1790129 (PMC13045876; doi:10.3389/fpls.2026.1790129)
Supplement: Supplementary file 1 [file DataSheet1.docx]

Supplementary Material

From laurels to parasites: The origin, evolution, systematics, and application of *Cassytha* (Lauraceae)

Zhi-Fang Liu^1,2,^*, Jie Li^3^, John G. Conran^4^

^1^Institute of Leisure Agriculture, Shandong Academy of Agricultural Sciences, Jinan 250100, China

^2^Shandong Engineering Research Center of Ecological Horticultural Plant Breeding, Jinan 250100, China

^3^Plant Phylogenetics and Conservation Group, Center for Integrative Conservation & Yunnan Key Laboratory for Conservation of Tropical Rainforests and Asian Elephants, Xishuangbanna Tropical Botanical Garden, Chinese Academy of Sciences, Mengla, Yunnan 666303, China

^4^Environment Institute, School of Biological Sciences, Adelaide University, North Terrace, Adelaide, SA 5000, Australia

**Literature Search Strategy:**

**1. Databases:** The primary literature search was conducted using the following electronic databases: **Web of Science Core Collection, Scopus, and China National Knowledge Infrastructure (CNKI)**.

**2. Search Terms:** Key search terms (and their combinations) included: ***Cassytha***, **Lauraceae**, **parasitic plant**, **stem parasite**, **haustoria**, **evolution**, **phylogeny**,

**biogeography, alkaloids**, **phytochemistry**, and **biological control**. Both English and Chinese terms were used where applicable. **Time Frame:** The search aimed to capture literature from the earliest relevant records up to **January 2026**. Emphasis was placed on incorporating studies published after the 2022 review (The angiosperm stem hemiparasitic genus *Cassytha* (Lauraceae) and its host interactions: A review) by Zhang et al. to ensure the inclusion of recent advances.

**3. Screening Process:** The initial database searches yielded approximately **300 potentially relevant records**. Titles and abstracts were screened for relevance to the core themes of this review: **evolutionary origin, phylogenetic systematics, morphological adaptation, biogeography, medicinal phytochemistry, and ecological applications of *Cassytha***.

**4. Inclusion/Exclusion Criteria:**

- **Included:** Peer-reviewed original research articles, reviews, and authoritative monographs directly related to *Cassytha*’s evolution, taxonomy, ecology, chemistry, or applications. Key foundational papers on Lauraceae systematics and parasitic plant evolution were also included for context.
- **Excluded:** Duplicate records, non-peer-reviewed sources (e.g., theses, conference abstracts without full proceedings), and articles not accessible in full text or without reliable English/Chinese translations. Articles focused solely on other parasitic genera without substantive discussion of *Cassytha* were also excluded.
- **Final Selection:** The initial search and screening process identified nearly 300 relevant articles. After a full-text assessment for relevance and quality, and in consideration of the journal’s guidelines on reference number, we ultimately cited **a**pproximately 30 of the most pertinent references to construct a focused, up-to-date synthesis that addresses the specific aims outlined in our introduction.
